# Use of Human Dental Pulp and Endothelial Cell Seeded Tyrosine-Derived Polycarbonate Scaffolds for Robust *in vivo* Alveolar Jaw Bone Regeneration

**DOI:** 10.3389/fbioe.2020.00796

**Published:** 2020-07-17

**Authors:** Weibo Zhang, Shruti Saxena, Amir Fakhrzadeh, Sara Rudolph, Simon Young, Joachim Kohn, Pamela C. Yelick

**Affiliations:** ^1^Department of Orthodontics, Division of Craniofacial and Molecular Genetics, Tufts University School of Dental Medicine, Boston, MA, United States; ^2^New Jersey Center for Biomaterials, Rutgers University, Piscataway, NJ, United States; ^3^Department of Oral and Maxillofacial Surgery, The University of Texas Health Science Center at Houston School of Dentistry, Houston, TX, United States

**Keywords:** dental pulp stem cells, alveolar bone regeneration, tyrosine-derived polycarbonate scaffolds, bone remodeling, craniomaxillofacial bone regeneration

## Abstract

The ability to effectively repair craniomaxillofacial (CMF) bone defects in a fully functional and aesthetically pleasing manner is essential to maintain physical and psychological health. Current challenges for CMF repair therapies include the facts that craniofacial bones exhibit highly distinct properties as compared to axial and appendicular bones, including their unique sizes, shapes and contours, and mechanical properties that enable the ability to support teeth and withstand the strong forces of mastication. The study described here examined the ability for tyrosine-derived polycarbonate, E1001(1K)/β-TCP scaffolds seeded with human dental pulp stem cells (hDPSCs) and human umbilical vein endothelial cells (HUVECs) to repair critical sized alveolar bone defects in an *in vivo* rabbit mandible defect model. Human dental pulp stem cells are uniquely suited for use in CMF repair in that they are derived from the neural crest, which naturally contributes to CMF development. E1001(1k)/β-TCP scaffolds provide tunable mechanical and biodegradation properties, and are highly porous, consisting of interconnected macro- and micropores, to promote cell infiltration and attachment throughout the construct. Human dental pulp stem cells/HUVECs seeded and acellular E1001(1k)/β-TCP constructs were implanted for one and three months, harvested and analyzed by micro-computed tomography, then demineralized, processed and sectioned for histological and immunohistochemical analyses. Our results showed that hDPSC seeded E1001(1k)/β-TCP constructs to support the formation of osteodentin-like mineralized jawbone tissue closely resembling that of natural rabbit jaw bone. Although unseeded scaffolds supported limited alveolar bone regeneration, more robust and homogeneous bone formation was observed in hDPSC/HUVEC-seeded constructs, suggesting that hDPSCs/HUVECs contributed to enhanced bone formation. Importantly, bioengineered jaw bone recapitulated the characteristic morphology of natural rabbit jaw bone, was highly vascularized, and exhibited active remodeling by the presence of osteoblasts and osteoclasts on newly formed bone surfaces. In conclusion, these results demonstrate, for the first time, that E1001(1K)/ β-TCP scaffolds pre-seeded with human hDPSCs and HUVECs contributed to enhanced bone formation in an *in vivo* rabbit mandible defect repair model as compared to acellular E1001(1K)/β-TCP constructs. These studies demonstrate the utility of hDPSC/HUVEC-seeded E1001(1K)/β-TCP scaffolds as a potentially superior clinically relevant therapy to repair craniomaxillofacial bone defects.

## Introduction

The most common cause for craniomaxillofacial (CMF) bone damage is acute trauma, which can result in serious health problems with respect to both the physical and psychological well-being of civilians and military personnel (Grayson et al., 2015; Gaihre et al., 2017). Craniomaxillofacial injuries represent up to 26% of all battlefield injuries, as occurred in Operation Iraqi Freedom/Operation Enduring Freedom (Afghanistan)^1^ (Lew et al., 2010; U.S. Army Medical Research and Development Command, 2019). Craniofacial defects are also a common birth defect (1:700), which poses significant challenges for the health and development of, and reparative therapies for, affected children whose facial bones are actively growing (Caballero et al., 2017). Large CMF boney defects caused by tumor resection, trauma, and birth defects commonly require highly specialized surgical interventions due to the limited regenerative potential of craniofacial bones. As such, clinical approaches to repair craniofacial bone reconstruction remain quite challenging. As for other bone defect repair therapies, autogenous bone grafting remains the gold standard, not only because autogenous bone grafts possess highly desirable properties including osteoconduction, osteoinduction, osteogenesis, and structural support of the autogenous bone graft (Fillingham and Jacobs, 2016), but also due to their superior immogenic and biocompatibility (Younger and Chapman, 1989). Disadvantages to autologous bone grafting include limited availability of donor tissue, donor site morbidity and variable bone graft survival (Jakoi et al., 2015). Although xenograft and allograft therapies are also commonly used, clinical applications for these approaches are limited due to concerns about potential immune rejection and often inadequate bone regeneration.

To date, tissue engineering approaches using natural bone forming cells have provided the most promising therapies for effective boney defect repair (Ai et al., 2012; Polymeri et al., 2016). With respect to cell contributions for CMF repair therapies, it is important to remember that craniofacial bones are neural crest cell (NCC) derived, while axial and appendicular bones are mesodermal derived (Chai and Maxson, 2006). As such, CMF bones, and the cells that form them, exhibit unique responses to developmental, mechanical and homeostatic stimuli (Sodek and McKee, 2000). Moreover, many craniofacial bones undergo intramembranous rather than endochondral ossification used to form long bones (Karaplis, 2002). For applications in CMF repair, craniofacial bone grafts have shown superior volumetric bone maintenance and survival as compared to other commonly used bone sources such as rib, tibia, or iliac crest. Bone grafts of mesoderm origin were replaced by fibrous tissue in 6–8 months, while grafts harvested from vomer, nasal, or ethmoid bones retained their normal bony structure for up to 5 years (Sullivan and Szwajkun, 1991). Bone marrow-derived mesenchymal stem cells (BMSCs) isolated from mesoderm-origin iliac crest are another commonly used cell type used in cell-based therapies for craniofacial bone regeneration (Filho Cerruti et al., 2007; Hasani-Sadrabadi et al., 2020).

Autologous grafts containing live bone marrow-derived cells have also been used in the clinic to repair craniofacial defects (Marcacci et al., 2007; Macchiarini et al., 2008; Mehrabani et al., 2018). Human mandibular or maxillary BMSCs demonstrated increased cell proliferation, delayed senescence, and stronger expression of osteoblastic markers as compared to iliac-crest-derived marrow cells from the same patients (Akintoye et al., 2006), suggesting distinct functions and differentiation potential. Another study showed that mandible derived BMSCs demonstrated augmented alkaline phosphatase activity, mineralization, and osteoblast gene expression (Aghaloo et al., 2010). Still, the relatively small size and anatomical complexity of the maxilla and mandible make successful autologous bone cell harvest quite challenging, and great care must be taken not to damage the harvest site. In contrast, NCC derived dental pulp stem cells harvested from extracted wisdom and other teeth, have been shown to form mineralized tissues exhibiting characteristics of both alveolar bone and dentin (Young et al., 2002, 2005; Duailibi et al., 2004; Iohara et al., 2006; Zhang et al., 2006, 2008; Abukawa et al., 2009). The fact that human DPSCs (hDPSCs) are of NCC origin, and are easily isolated from otherwise discarded teeth, combined with their demonstrated utility to effectively regenerate craniofacial mineralized tissues, makes them a highly suitable cell source for craniofacial and dental tissue regenerative therapies in humans.

Another important consideration for Tissue Engineered and Regenerative Medicine therapies is the choice of scaffold to use. In the study described here, we use Tyrosine-derived Polycarbonate (TyrPCs) scaffolds, recently developed by the Kohn laboratory, which have been extensively characterized for applications as stents, drug-delivery devices, bone pins, and calvarial and long bone regeneration scaffolds (Kim et al., 2011). To date, TyrPC family derived porous scaffolds fabricated from 90 mol% desaminotyrosyl-tyrosine ethyl ester (DTE), 10 mol% desaminotyrosyl-tyrosine (DT), and 1 mol% poly(ethylene glycol) (PEG) with 1 kDa molecular weight, abbreviated as E1001(1k), have been shown to support robust bone regeneration in rabbit critical-sized calvarial and long bone defect repair models, particularly when the E1001(1k) scaffolds contained calcium phosphate (Kim et al., 2012, 2015; Guda et al., 2014). Based on promising results using E1001(1k) scaffolds for calvarial bone tissue regeneration, we further demonstrated successful jaw bone regeneration using hDPSC-seeded E1001(1k)/β-TCP scaffolds in a small animal, rat ramus defect repair model (Zhang et al., 2016).

In this study, to facilitate moving this approach to clinical relevance, we have modified and improved upon our defect repair model in the following manner. We have dramatically scaled-up the size of our constructs more than 20×, from a 5 × 1 mm disk (19.63 mm^3^) to an 10 × 6 mm cylinder (471 mm^3^). Next, we have tested these large constructs using a new, full thickness mandible and tooth defect repair model, in a medium sized rabbit animal model. Lastly, we have added human umbilical vein endothelial cells (HUVECs) to our model to improve vascularized bone formation. The results of this study, described below, demonstrate that hDPSC/HUVEC seeded E1001(1k)/β-TCP scaffold constructs support the regeneration of highly vascularized alveolar jaw bone that exhibits actively remodeled bone formation. These promising results validate the utility of hDPSC/HUVEC seeded E1001(1k)/β-TCP scaffolds as a potential new and effective therapy for repairing CMF defects.

## Materials and Methods

### Scaffold Fabrication

Cylindrical, porous E1001(1k)/β-TCP scaffolds were fabricated as previously described (Pulapura and Kohn, 1992). Briefly, 1.20 g of E1001(1k) polymer (molecular weight: 270 kDa) was dissolved in 0.84 mL of deionized water and 5.16 mL of 1,4-dioxane overnight. The polymer solution was then homogeneously mixed with 10.80 g of sodium chloride (NaCl) porogen (200–400 μm particle size) and 0.51 g of β-TCP particles. The mixture was poured into a Teflon dish, quenched in liquid nitrogen and then freeze-dried. Cylindrical scaffolds (10 mm diameter × 6 mm thickness) were then punched out, leached in DI water, and dried in a lyophilizer. All the scaffolds were ethylene oxide sterilized and stored at -20°C until use. Micro-computed tomography (Micro-CT) and Scanning Electron Microscopy (SEM) were used to characterize and validate the surface structure of the fabricated scaffolds as published by us (Saxena et al., 2020).

### Cell Culture and Construct Fabrication

Two types of cells were used in this study – hDPSCs to regenerate alveolar bone tissue, and HUVECs to facilitate vascularization of the construct once implanted *in vivo*. hDPSCs were harvested and characterized as previously described by us (Zhang et al., 2011). Briefly, human teeth were extracted by trained clinicians at the Tufts University School of Dental Medicine using Tufts University IRB approved protocols. Dental pulp was then harvested, minced into small pieces, and digested using 0.4 mg/mL collagenase type I (Sigma-Aldrich, St. Louis, MO, United States) and 0.2 mg/mL dispase (Boehringer Mannheim, Indianapolis, IN, United States), and filtered to generate single cell suspensions. hDPSCs were expanded via *in vitro* cell culture in 5% CO_2_ at 37°C in dental mesenchymal medium with DMEM/F12 (Fisher, Hampton, NH, United States), 10% FBS (Sigma-Aldrich), 1% GlutaMAX (Fisher), 50 μg/mL ascorbic acid (Sigma-Aldrich), and 1% penicillin/streptomycin/amphotericin (PSA, Fisher), and then cryopreserved until use. The osteogenic and neurogenic differentiation potential of hDPSC lines was confirmed prior to use. The HUVEC cell line was purchased from ATCC (PSC100010, Manassas, VA, United States), expanded in vascular basal media (PCS100030, ATCC) with vascular endothelial growth factor (VEGF) growth kit (PCS100041, ATCC) in 5% CO_2_ at 37°C, and cryopreserved at passage three. Cryopreserved hDPSCs and HUVECs were thawed and expanded *in vitro* immediately prior to use. Equal numbers (1:1) of hDPSCs and HUVECs were seeded dynamically onto E1001(1k)/β-TCP scaffolds for a final density of 0.25 × 10^5^ cells/mm^3^. Cell-seeded and unseeded acellular scaffolds were *in vitro* cultured in 1:1 dental pulp and HUVEC medium with osteogenic supplements [100 nM dexamethasone (Sigma-Aldrich), 10 mM beta-glycerolphosphate (Sigma-Aldrich), and 50 μg/mL ascorbic acid (Sigma-Aldrich)] for one week prior to *in vivo* implantation. To demonstrate hDPSC and HUVEC attachment throughout E1001(1k)/β-TCP scaffolds at the time of implantation, two cell seeded constructs were embedded in Optimal Cutting Temperature compound (OCT, Sakura Finetek USA Inc, Torrance, CA, United States), cryosectioned, stained with phalloidin, and analyzed using an M2-Bio Zeiss fluorescent microscope (Zeiss, Germany).

### Rabbit Mandible Defect Repair Model

The rabbit mandible defect repair model used in this study was performed on New Zealand White Rabbits (3.5–4.5 kg) (Kim et al., 2012; Shah et al., 2016). All animal experiments were conducted under the guidance and approval of the Institutional Animal Care and Use Committee of Tufts University. A single construct implant was placed in the left side mandible of each of 10 rabbits, including three cell-seeded and 2 acellular constructs for each of two time points, at 1 and 3 months. Briefly, fully anesthetized rabbits were placed in a dorsal position, and an incision was made from the mentum to the midpoint between left and right mandibular angles. Careful dissection of the fascia and muscle was performed to expose the buccal cortical plate of the mandible in the region of the premolars. A full thickness mandibular bone defect centered on the second molar was then created using a 10 mm trephine bur, under constant saline irrigation. Buccal cortex bone, exposed tooth roots, and lingual cortex bone were sequentially removed to create a full thickness defect. The defect site was then thoroughly irrigated with sterile saline to remove any remaining bone and tooth fragments, and a cell seeded or acellular construct was placed into the defect. Muscle and fascia were then closed over the implant using 4–0 Vicryl suture, and the skin was closed with subcuticular stitches with 4–0 Vicryl. Heart rate, oxygen saturation, carbon dioxide, respiratory rate, and body temperature were monitored carefully throughout the procedure. In order to prevent fracture of the operated mandible, soft, Critical Care diet was provided for 2 weeks post-operation, followed by regular rabbit chow. After 1 and 3 months, implants and control unoperated hemi-mandibles were harvested using perfusion. The harvested mandibles were then re-fixed in 4% formalin and processed for subsequent analyses.

### Evaluation of Bioengineered Mandibular Bone Implants

#### Micro-Computed Tomography Analyses

Bioengineered mandibular bone was analyzed using micro-CT and histological analyses. Harvested mandibular implants were assessed for new bone formation using a micro-CT imaging system (Skyscan 1176, Bruker MicroCT, Billerica, MA, United States). Scans were performed on all harvested implants and unoperated control jaws at a spatial resolution of 9 μm, together with two control rods with BMD values of 0.25 and 0.75 g/cm^3^ CaHA. Micro-CT data was then reconstructed using NRecon software (Bruker Micro-CT). A full thickness, 10 mm diameter cylindrical area that matched the defect area was further selected and evaluated for new bone regeneration. Properties of the newly formed bone, including density of newly formed bone, total bone volume (BV) and bone volume/tissue volume (BV/TV) were fully characterized using Avizo (Version 1.6.9.15, ThermoFisher Scientific, Materials & Structural Analysis Division, Hillsboro, OR, United States) and CTAn (Bruker Micro-CT).

#### Histological and Immunohistochemical Analyses

After Micro-CT analysis, the implants and control bone samples were decalcified in 1:1 solution of 45% Formic acid (Sigma-Aldrich): 20% sodium citrate (Sigma-Aldrich), dehydrated, embedded in paraffin, and sectioned at 7 μm intervals. The sections were then deparaffined, and histological stained using Hematoxylin and Eosin (H&E) or Masson’s Trichrome staining. Immunofluorescent analyses were performed on the deparaffined sections using following antibodies for the odontoblast differentiation marker Dentin sialophosphoprotein (DSPP, abx176139, Abbexa Ltd, Cambridge, CB4 0EY, United Kingdom), the bone marker Osteocalcin (OC, kind gifts of Dr. Jaro Sodek), the endothelial cell marker Factor VIII (ab61910, Abcam, Cambridge, United Kingdom), and the neuronal markers Nestin (ab18102, Abcam) and CLPP (sc-271284, Santa Cruz, Dallas, TX, United States). The slides were imaged using Zeiss Axiophot microscope and digital Zeiss Axiocam camera (Carl Zeiss AG, Jena, Germany).

#### Statistical Analyses

Statistically analysis on micro-CT results, including density of newly formed bone, BV, and BV/TV was performed by one-way ANOVA.

## Results

### Scaffold Fabrication

**FIGURE 1:**
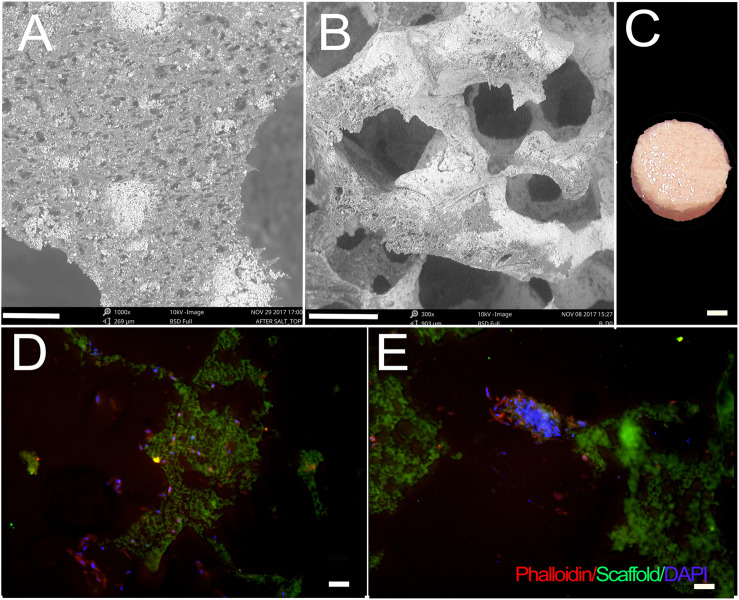
hDPC-seeded E1001(1k)/β-TCP scaffolds. **(A,B)** SEM imaging demonstrates highly organized and interconnected macro- and micro-pore structure of E1001(1k)/β-TCP scaffolds. **(C)** hDPSC/HUVEC seeded scaffolds after 1week *in vitro* culture in osteogenic media. **(D,E)** Phalloidin stained hDPSCs and HUVECs were detected throughout macro- and micro-pores of 1-week cell-seeded *in vitro* cultured constructs. Cytoskeletal F-Actin was stained using Alexa Fluor 594-conjugated phalloidin (red). Nuclei were stained with DAPI (blue). E1001(1k)/β-TCP scaffolds exhibited autofluorescence under GFP filter (green). Scale bars = 80 μm **(A)**, 200 μm **(B)**, 2 mm **(C)** and 100 μm **(D,E)**.

Fabricated scaffolds were validated using micro-CT and SEM (Saxena et al., 2020; Figure 1). Using SEM, E1001(1k)/β-TCP scaffolds exhibited high porosity, and a bimodal distribution of micropores and macropores that resembled the pore size range and architecture of natural trabecular bone. The highly organized microstructure exhibited interconnected and open pore architecture (Figures 1A,B). None of the cell-seeded or acellular E1001(1k)/β-TCP scaffolds showed any noticeable morphological changes after one-week *in vitro* culture in osteogenic media (Figure 1C). Replicate constructs were analyzed at the time of implantation via paraffin embedding and sectioning, which revealed phalloidin stained cells throughout the construct that showed good attachment and spreading throughout the construct (Figures 1D,E).

### Surgical Outcomes

**FIGURE 2:**
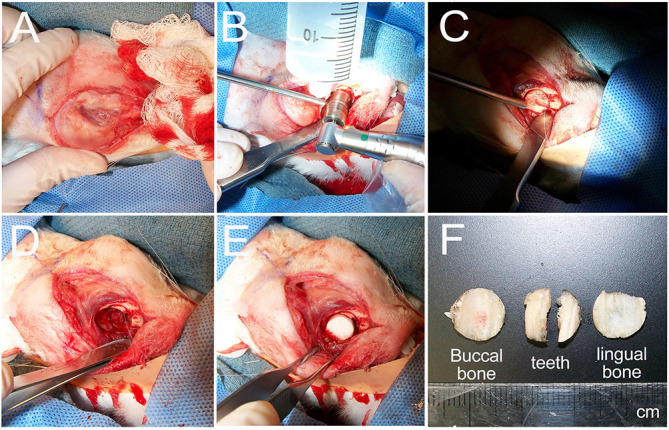
Rabbit mandible defect repair model. **(A)** An incision was made from the mentum to the midpoint between left and right mandibular angles. **(B)** A 10-mm trephine bur was used to drill evenly through the buccal cortex. **(C)** The buccal cortex was removed using a periosteal elevator. **(D)** A full thickness defect was created by removal of buccal cortex, tooth roots and lingual cortex. **(E)** Constructs were then placed into the prepared defect. **(F)** Example of removed buccal jaw bone, tooth roots and lingual jaw bone.

Surgeries were performed successfully on all 10 rabbits (Figures 2A–E). Reproducible full thickness, mandible and tooth root surgical defects were created using a 10-mm trephine bur with continuous irrigation. No other defects were observed (Figures 2B–D,F). The buccal bone, tooth roots, and lingual bone were removed without incident (Figure 2F). All operated rabbits showed good mucosal wound healing within 7 days of implant surgery, and no weight loss or other adverse reactions to the surgical procedure were observed in any of the host rabbits. After 1 and 3 months, no noticeable changes in the dentition or jaw bones was observed at any of the implant sites relative to the contralateral unoperated control mandible side.

### Micro-CT Analyses of Harvested Implants

**FIGURE 3:**
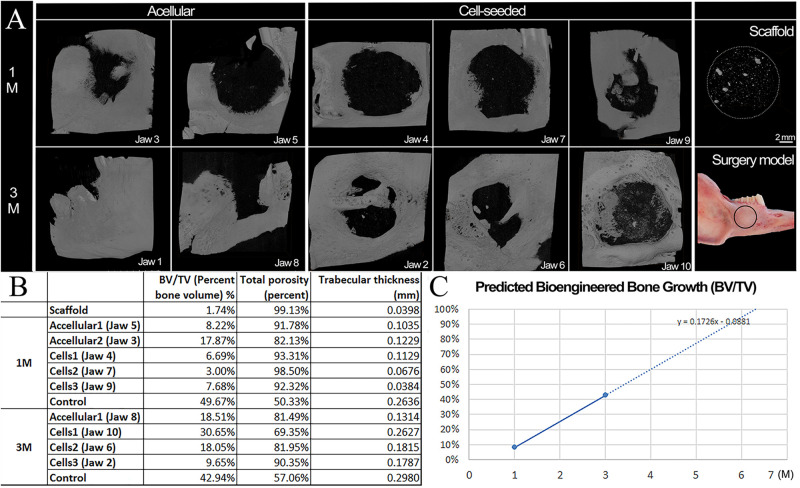
Microcomputed tomography (Micro*−*CT) analyses of harvested implants. **(A)** Representative Micro-CT images of bioengineered bone in harvested 1 and 3 month acellular and cell-seeded implants. Micro-CT image of a E1001(1k)/β-TCP scaffold alone. Significantly more calcified tissue formation was observed in 3 month implants as compared to 1 month implants. Please note that acellular Jaw 1 showed unwanted tooth eruption into the defect site, and subsequent high mineralized tissue formation. The cell-seeded scaffolds exhibited more homogeneous mineralized tissue formation throughout the implants as compared to acellular constructs. The black circle on the Surgery Model panel indicates the defect area. **(B)** Mineralized tissue formation in the defect site was quantified by bone volume/tissue volume (BV/TV), total porosity and average trabecular thickness as measures of bioengineered bone maturity. **(C)** Predicted time to full healing was estimated to be 6 months *in vivo* implantation. Scale bars = 2 mm **(A,B)**. PM2, premolar 2.

Three dimensional (3D) Micro-CT analyses of harvested implants showed an easily identifiable radiolucent circular defect site in all harvested mandibles at 1 and 3 months (Figure 3 and Supplementary Videos S1–S6). Radiopaque areas in the defect site indicated bioengineered mineralized tissue formation (Figure 3A). Comparatively more mineralized tissue formation was observed in constructs implanted for 3 months as compared to those implanted for 1 month (Figure 2A). hDPSC-HUVEC seeded implants exhibited more uniform calcified tissue formation throughout the entire implant as compared to acellular construct implants (Figure 3A). Rabbit host tooth growth into the implant was observed in two of the acellular 3 month implants, but not in any of the other 1 or 3 month implants. We believe this was due to the fact that the tooth roots were not sufficiently damaged in these two animals, and so continued to erupt in these animals and not in the others.

Bioengineered new hard tissue formation was quantified by bone BV/TV measurements, and by measuring total porosity within the selected defect site area using Micro-CT image analyses. Trabecular thickness distribution was also quantified to evaluate the maturity of newly formed bone (Figure 3B). Both measurements showed that cell seeded constructs exhibited increased BV and increased trabecular thickness (i.e., maturity) over time, as compared to acellular implants. A linear growth plot of BV/TV measurements at 1 and 3 months was used to predict that the full regeneration of bioengineered mandibular bone that matched that of the natural rabbit mandible would occur after ∼6 months *in vivo* implantation (Figure 3C). No specific trend was observed on bone density (BD) or BV among samples and controls (Raw data present in Supplementary Table S1).

Importantly, the bioengineered jaw bone adopted the shape and contours of the natural rabbit mandibular bone. As evident in Supplementary Videos of reconstructed micro-CT imaging, bioengineered bone that formed on cell-seeded E1001(1k)/β-TCP scaffolds exhibited many of the unique features of the natural rabbit mandible, including rounded contours and depressions, and a wing of bone that formed a horizontal ridge across the defect, particularly evident in hDPSC-seeded 3 month implants of jaws 2 and 6 (Figure 3 and Supplementary Video S3). Importantly, these results indicate that bioengineered mandibular bone exhibited active remodeling in response to the mechanical forces of chewing. However, no significant difference was found with respect to bioengineered BD, BV, or BV/TV, between the cell-seeded and acellular groups, likely due to the limited number of samples, and the fact that in both of the 3-month acellular samples, teeth continued to grow into the defect site.

### Histological Analyses of Bioengineered Bone Constructs

**FIGURE 4:**
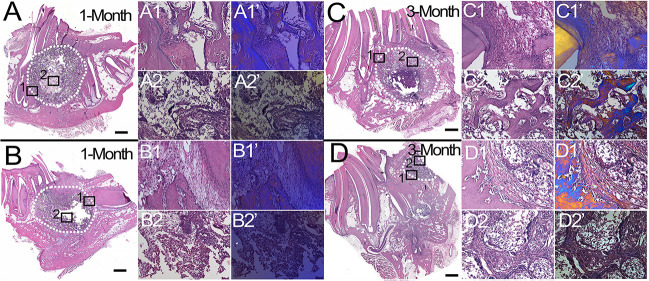
Histological analysis of mandibular bone defect healing. H&E stained coronal sections of harvested rabbit mandibular implants. After 1 month implantation, no obvious difference was observed between cell-seeded **(A)** and acellular **(B)** implants. After 3 months implantation, cell-seeded samples exhibited robust new bone formation throughout the defect area **(C)**, while no obvious bone formation was evident in the center area of acellular implants **(D)**, (compare **C2,C2’** versus **D2,D2’**). Dotted white lines indicate the defect area **(A–D)**. Panels **(1,1’)** and **(2,2’)** are high magnification images of boxed areas of low mag panels **(A–D).** Panels **(1’,2’)** are corresponding polarized light images, which show robust bone formation in cell-seeded 3 month implant **(C2’)** and not in acellular 3 month implant **(D2’)**. Scale bar = 1 mm **(A–D)**, and 50 μm **(1,2,1’,2’)**.

**FIGURE 5:**
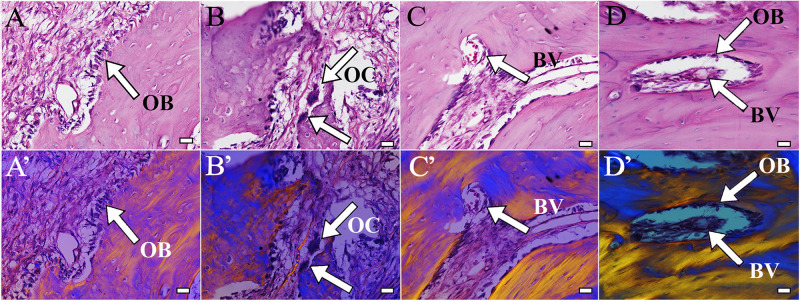
Bioengineered bone exhibits active remodeling. Bioengineered bone **(A–C)** showed typical structure of natural rabbit jaw bone **(D)**. Active remodeling was indicated by presence of osteoblasts (**A**, arrow) and osteoclasts (**B**, arrow). Bioengineered bone was highly vascularized and contained bone marrow (**C**, arrow), similar to natural rabbit jaw bone and bone marrow after 3 months implantation [9 months of age **(D)**]. **(A’–D’)** Polarized light images of panels **(A–D)**, respectively. Scale bar = 25 μm **(A–C)**. OB, osteoblasts; OC, osteocalcin; BV, blood vessel.

H&E staining of coronally sectioned harvested implants was used to assess new bioengineered bone formation at the defect site. For 1 month implanted harvested constructs, no obvious differences in bioengineered bone formation were observed between cell-seeded versus acellular implants (Figure 4A vs. 4B). In contrast, after 3 months implantation, cell-seeded constructs showed robust bone formation throughout the entire defect area while no obvious bone formation was found in the center of the acellular construct implants (Figure 4C vs. 4D). Active remodeling of bioengineered bone was evident by the presence of osteoblasts, osteoclasts, and osteocytes, respectively (Figures 5A–C), similar to those observed in contralateral control natural rabbit jaw bone (Figure 5D). Bioengineered bone exhibited highly organized mineralized tissue formation as revealed by polarized light imaging (Figures 5A’–C’), similar to that of contra-lateral natural rabbit jaw bone (Figure 5D’). Finally, bioengineered mandibular rabbit bone exhibited many typical features of natural bone, including robust vascularization (Figure 5C, arrow).

**FIGURE 6:**
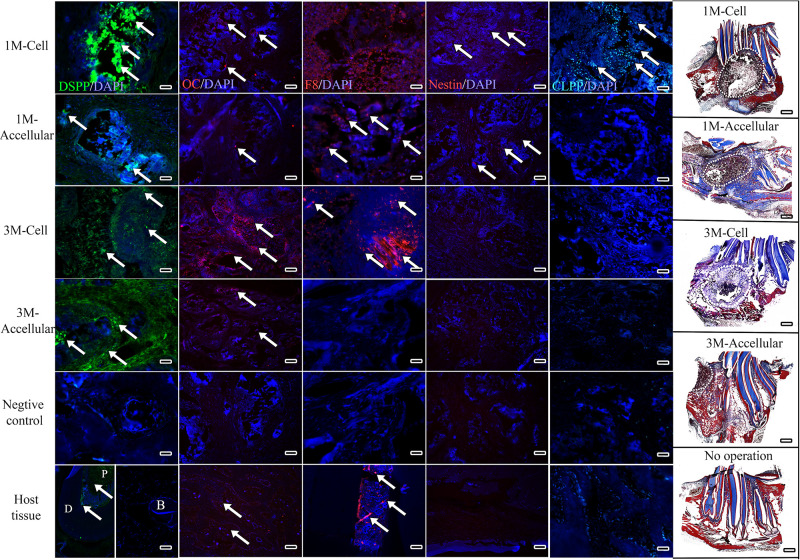
Bioengineered bone exhibits robust differentiation marker expression. Representative images from the center of sectioned implants showed positive DSPP (green) expression in all harvested implants, with strongest expression in 3 month cell-seeded implants. OC expression (red) also appeared strongest in 3 month cell-seeded implants. Faint F8 expression (red) indicated the presence of HUVECs and organized blood vessel formation (arrows). Nestin expression (red) was detected in 1 month and not in 3 month harvested implants. CLPP positive human DPSCs/HUVECs (blue–green) were only detected in 1-month hDPSC/HUVEC seeded implants. White dots outline the implant site in Mason’s Trichrome stained sections (far right panels). Scale bar = 50 μm in IF panels and 1mm in Mason’s Trichrome panels.

Immunofluorescent staining, performed to assess expression of dentin (DSPP), bone (OC), and angiogenic (F8) markers, also indicated robust bioengineered bone formation. Positive DSPP expression was observed in all harvested implants at 1 and 3 months, with much stronger expression observed in cell-seeded constructs, especially after 3-months implantation (Figure 6, arrows). OC exhibited strong expression in 3-month implants, particularly in cell-seeded constructs. Scattered expression of F8 indicated the presence of seeded HUVECs and the formation of organized and functional blood vessels (Figure 6, F8, red). Finally, Nestin positive hDPSCs and CLPP positive hDPSC/HUVECs were only detected in 1-month cell seeded constructs, indicating that human cells were no longer detectable by 3 months implantation (Figure 6).

## Discussion

The studies described here demonstrate the potential for hDPSC-HUVEC seeded E1001(1k)/β-TCP constructs as a potentially new and improved therapy to repair CMF defects. One innovative aspect of this study is the use of Tyrosine Polycarbonate derived scaffolds for robust bone formation. The ability to modulate the amount of the three monomers used to fabricate these scaffolds – DTR, DT, and PEG – allows for the precise control of physical, chemical, biomechanical, degradative, and biological properties of the scaffold (Pulapura and Kohn, 1992; Bourke and Kohn, 2003). The formulation used in the studies described here, E1001(1k) derived porous scaffolds fabricated from 90 mol% DTE, 10 mol% DT, and 1 mol% PEG with 1 kDa molecular weight, were shown to support robust bone regeneration in calvarial and long bone defect repair models, particularly when the E1001(1k) scaffolds contained calcium phosphate (Kim et al., 2012, 2015; Guda et al., 2014). Based on these promising results, our lab has tested E1001(1k)/β-TCP scaffolds seeded with NCC-derived-hDPSC in a small animal, rat ramus defect repair model, which showed successful jaw bone regeneration (Zhang et al., 2016). Therefore, the objective of this study was to test hDPSC-seeded E1001(1k)/β-TCP scaffolds on a more clinical-related large scale rabbit critical sized mandibular defect repair model.

Although progress has been made in developing improved surgical techniques and in clinical outcomes for skeletal defect repair, craniofacial bone regeneration therapies remain quite challenging, partly due to the limited availability of autologous CMF tissue sources (Amini et al., 2012). Unique characteristics of mandibular versus axial/appendicular skeletal bone have been demonstrated in ovariectomized rodent models, where ovariectomy and malnutrition models showed that the mandible lost significantly less bone than the proximal tibia (Miller et al., 1997; Mavropoulos et al., 2007). Similar to successful skeletal bone regeneration therapies, biomimetic CMF bone grafts should exhibit high biocompatibility, mechanical properties that support craniofacial structure and function during the bone healing process, and be easily and accurately shaped to precisely fit the uniquely complex geometries of craniofacial bones (Antonov et al., 2004; Popov et al., 2004).

Many different types of materials have been used to facilitate craniofacial reconstructions, such as titanium (Kuttenberger and Hardt, 2001), calcium phosphate (Ho et al., 2011; Thrivikraman et al., 2017), and synthetic polymers (Cohen et al., 2004). In general, 3D biomimetic scaffolds that exhibit physical properties closely resembling those of the target tissue were found to provide the best outcomes. For craniofacial bone reconstructions, optimal scaffolds would exhibit Haversian-like structures present in natural bone and pore diameter of ∼100 μm (Hutmacher, 2000). The E1001(1k)/β*-*TCP, porous scaffolds used here consist of an interconnected bimodal pore structure, with 200–400 μm diameter macropores that allow for cellular infiltration and bone formation, interconnected with ∼20 μm diameter micropores that facilitate oxygen and nutrient diffusion, and induce bone-like apatite formation (Saiz et al., 2013). We observed mineralized tissue formation both within and on the surfaces of hDPSC seeded and unseeded E1001(1k)/β-TCP scaffolds, further demonstrating the superior osteoconductivity of these scaffolds. Indeed, the ability to direct new bone formation in acellular scaffold implants demonstrates that E1001(1k)/β-TCP scaffolds can efficiently recruit host cells to participate in mineralized tissue regeneration.

Micro*-*CT and histological analyses showed that implanted hDPSC/HUVEC-seeded E1001(1k)/β-TCP scaffolds exhibited a unique pattern of mineralized tissue formation as compared to acellular scaffolds. Fewer but larger areas of calcified tissue formed within acellular scaffold implants, while broader areas of smaller, homogeneously and evenly distributed new bone formed throughout hDPSC/HUVEC seeded implants. Importantly, after 3 months implantation, we found that the bioengineered mandibular bone exhibited many of the unique features and three dimensional contours of natural rabbit jaw bone, particularly evident in the 3 month cell seeded Micro-CT video reconstructions (Supplementary Video S3). Although we found no statistical differences between the our cell seeded and acellular constructs with respect BD, BV, and BV/TV, the cell-seeded groups exhibited much more evenly distributed new bone formation throughout the entire implant, similar to what we have observed in our prior rat ramus repair model (Zhang et al., 2016). Most importantly, the bioengineered bone demonstrated active remodeling only ion the hDPSC/HUVEC seeded samples. Together, these promising results indicate that hDPSC/HUVEC seeded E1001(1k)/ β-TCP scaffolds exhibit active remodeling during jaw bone regeneration, and are responsive to mechanical forces that guide proper size and shape of CMF bones.

Furthermore, the expression of DSPP, a dentin specific matrix protein normally expressed in naturally formed alveolar bone, was only observed in bioengineered bone derived from hDPSC-seeded scaffold implants, and not in acellular scaffolds implants. Together, these results suggest that E1001(1k)/β-TCP scaffolds exhibited the ability to support hDPSC differentiation and the formation of mineralized bone tissue resembling that of natural jaw bone. We further demonstrated that CLPP expressing hDPSCs were detectable in hDPSC/HUVEC seeded implants harvested at 1 month, but not at 3 months. These results are consistent with numerous reports showing that implanted human cells contribute to long term tissue regeneration, but do not maintain long term residence in the implants (Dupont et al., 2010).

During bone formation and repair, osteogenesis and angiogenesis are tightly coupled processes (Hsiong and Mooney, 2006; Clarkin and Gerstenfeld, 2013). Blood vessels not only carry oxygen and nutrients to the developing bone, but also play an active role in bone formation and remodeling by mediating interactions between osteoblasts, osteocytes, osteoclasts and endothelial cells (Athanasopoulos et al., 2007; Wang et al., 2007). For the study described here, we seeded 10 mm diameter × 6 mm thick E1001(1k)/β-TCP scaffolds with both HUVECs and hDPSCs, to facilitate angiogenesis and alveolar bone formation. Our analyses showed significant blood vessel formation throughout the implants, especially after 3-months implantation. Some signs of necrosis were observed in the center of some of the harvested implants, suggesting the need for improved methods to promote blood vessel formation, such as the use of VEGF.

In addition to their osteo/odontogenic differentiation capabilities, hDPSCs exhibit highly desirable immunomodulatory properties, making them a promising therapeutic cell source for clinical use (Andrukhov et al., 2019; Hossein-Khannazer et al., 2019). Multiple studies have reported that no clinical or histological rejection of hDPSCs when implanted in animals without immunosuppression (de Mendonca Costa et al., 2008; Fernandes et al., 2018). Similarly, in this study we found no noticeable host rejection from the hDPSC/HUVEC seeded constructs.

In summary, our long-term goal is to develop improved therapies to effectively repair human CMF defects using bioengineered alveolar jawbone, and eventually bioengineered jaw bone and teeth constructs. Tissue Engineering-based CMF regeneration therapies have the potential to significantly improve patient outcomes and reduce surgical costs. Since CMF injuries account for ∼80% of all battlefield injuries, these studies address an important medical need for both military personnel and civilians afflicted with birth defects, trauma and surgical resection (Lew et al., 2010). The findings presented here, significantly expand upon our prior findings by demonstrating that scaled up constructs can be used to effectively repair a fill sized mandible and tooth defect in a medium sized animal model. which demonstrate the successful regeneration of vascularized craniofacial bone using easily accessible human dental pulp stem cells and HUVECs combined with novel E1001(1k)/β-TCP scaffolds, provide a potentially new and more effective, clinically relevant therapy to repair CMF defects.

## Data Availability Statement

The datasets generated for this study are available on request to the corresponding author.

## Ethics Statement

The animal study was reviewed and approved by Tufts IACUC Committee.

## Author Contributions

WZ made substantial contributions to the conception and design, acquisition, analysis and interpretation of data, and critical drafting and revising for important intellectual content. SS made substantial contributions to the conception and design, the acquisition, analysis or interpretation of data, and drafting and revising for important intellectual content. AF made substantial contributions to the conception and design of the work, and analysis and interpretation of data. SR made substantial contributions to the acquisition and analysis of data. JK made substantial contributions to the conception and design, analysis and interpretation of data. SY devised and perfected the new rabbit mandible defect repair model used in this study, visited Tufts University to participate in the surgeries, and to teach the technique to Yelick Lab members and Tufts Veterinary Staff. PY made substantial contributions to the conception and design, acquisition, analysis and interpretation of data, and drafting and revising it critically for important intellectual content. All authors provided approval for publication and agreed to be accountable for all aspects of the work in ensuring that questions related to accuracy or integrity are appropriately investigated and resolved.

## Conflict of Interest

The authors declare that the research was conducted in the absence of any commercial or financial relationships that could be construed as a potential conflict of interest.

## Footnotes

1 https://mrdc.amedd.army.mil/index.cfm/program_areas/medical_research_and_development/ccc_overview

## Supplementary Material

TABLE S1 Micro-CT analyses of Bone Density and Bone Volume showed no difference between accellular and cell seeded samples.

VIDEO S1 Micro-CT analysis of a **cell-seeded** construct implanted *in vivo* for one-month. The white radio opaque areas indicating the formation of calcified tissues can be observed throughout the implanted site.

VIDEO S2 Micro-CT analysis of an **acellular** construct implanted *in vivo* for one-month. The white radio opaque areas indicating the formation of calcified tissues can be observed throughout the implanted site.

VIDEO S3 Micro-CT analysis of a **cell-seeded** construct implanted *in vivo* for three months. Significant bone formation can be observed in the defect area, where highly calcified nodules are present throughout the implanted site.

VIDEO S4 Micro-CT analysis of an **acellular** construct implanted *in vivo* for three months. In this animal, continuous tooth eruption occurred through the implant site due to insufficient injury of the remaining tooth roots during implant surgery. Note that no distinct calcified bone nodules were found in the non-tooth containing defect area, indicating no obvious bone formation in acellular implant after 3 months.

VIDEO S5 Micro-CT analysis of an operated control rabbit jaw 3 months after surgery on contralateral side.

VIDEO S6 Micro-CT analysis of an E1001(1k)-βTCP scaffold alone, prior to cell culture.

## Abbreviations

BMSCs: bone marrow derived stromal cells
BV: bone volume
BV/TV: bone volume/tissue volume
CMF: craniomaxillofacial
DSPP: dentin sialophosphoprotein
DT: desaminotyrosyl-Tyrosine
DTE: desaminotyrosyl-tyrosine ethyl ester
hDPSCs: human dental pulp stem cells
H&E: hematoxylin and eosin
HUVECs: human umbilical vein endothelial cells
Micro-CT: micro-computed tomography
NCC: neural crest cell
OC: osteocalcin
OCT: optimal cutting temperature compound
PSA: penicillin/streptomycin/amphotericin
PEG: poly(ethylene glycol)
SEM: scanning electron microscopy
β -TCP: β -tricalcium phosphate;TyrPCs tyrosine-derived polycarbonate
VEGF: vascular endothelial growth factor.
