# Corrigendum: Use of Human Dental Pulp and Endothelial Cell Seeded Tyrosine-Derived Polycarbonate Scaffolds for Robust *in vivo* Alveolar Jaw Bone Regeneration

**DOI:** 10.3389/fbioe.2020.608275

**Published:** 2020-11-17

**Authors:** Weibo Zhang, Shruti Saxena, Amir Fakhrzadeh, Sara Rudolph, Simon Young, Joachim Kohn, Pamela C. Yelick

**Affiliations:** ^1^Department of Orthodontics, Division of Craniofacial and Molecular Genetics, Tufts University School of Dental Medicine, Boston, MA, United States; ^2^New Jersey Center for Biomaterials, Rutgers University, Piscataway, NJ, United States; ^3^Department of Oral and Maxillofacial Surgery, The University of Texas Health Science Center at Houston School of Dentistry, Houston, TX, United States

**Keywords:** dental pulp stem cells, alveolar bone regeneration, tyrosine-derived polycarbonate scaffolds, bone remodeling, craniomaxillofacial bone regeneration

Simon Young was unintentionally not included as an author in the published article. His affiliation is “Department of Oral and Maxillofacial Surgery, The University of Texas Health Science Center at Houston School of Dentistry, Houston, TX, United States.” The corrected Author Contributions statement appears below.

“WZ made substantial contributions to the conception and design, acquisition, analysis and interpretation of data, and critical drafting and revising for important intellectual content. SS made substantial contributions to the conception and design, the acquisition, analysis or interpretation of data, and drafting and revising for important intellectual content. AF made substantial contributions to the conception and design of the work, and analysis and interpretation of data. SR made substantial contributions to the acquisition and analysis of data. JK made substantial contributions to the conception and design, analysis and interpretation of data. SY devised and perfected the new rabbit mandible defect repair model used in this study, visited Tufts University to participate in the surgeries, and to teach the technique to Yelick Lab members and Tufts Veterinary Staff. PY made substantial contributions to the conception and design, acquisition, analysis and interpretation of data, and drafting and revising it critically for important intellectual content. All authors provided approval for publication and agreed to be accountable for all aspects of the work in ensuring that questions related to accuracy or integrity are appropriately investigated and resolved.”

In the original article, Shah et al. (2016) was not cited in the article. The citation has now been inserted in **Materials and Methods** section, **Rabbit Mandible Defect Repair Model**. The corrected paragraph appears below.

“The rabbit mandible defect repair model used in this study was performed on New Zealand White Rabbits (3.5–4.5 kg) (Kim et al., 2012, Shah et al., 2016). All animal experiments were conducted under the guidance and approval of the Institutional Animal Care and Use Committee of Tufts University. A single construct implant was placed in the left side mandible of each of 10 rabbits, including three cell-seeded and 2 acellular constructs for each of two time points, at 1 and 3 months. Briefly, fully anesthetized rabbits were placed in a dorsal position, and an incision was made from the mentum to the midpoint between left and right mandibular angles. Careful dissection of the fascia and muscle was performed to expose the buccal cortical plate of the mandible in the region of the premolars. A full thickness mandibular bone defect centered on the second molar was then created using a 10 mm trephine bur, under constant saline irrigation. Buccal cortex bone, exposed tooth roots, and lingual cortex bone were sequentially removed to create a full thickness defect. The defect site was then thoroughly irrigated with sterile saline to remove any remaining bone and tooth fragments, and a cell seeded or acellular construct was placed into the defect. Muscle and fascia were then closed over the implant using 4–0 Vicryl suture, and the skin was closed with subcuticular stitches with 4–0 Vicryl. Heart rate, oxygen saturation, carbon dioxide, respiratory rate, and body temperature were monitored carefully throughout the procedure. In order to prevent fracture of the operated mandible, soft, Critical Care diet was provided for 2 weeks post-operation, followed by regular rabbit chow. After 1 and 3 months, implants and control unoperated hemi-mandibles were harvested using perfusion. The harvested mandibles were then re-fixed in 4% formalin and processed for subsequent analyses.”

In the Acknowledgments, we missed to acknowledge Stacey Piotrowski. The corrected paragraph appears below.

“We thank all members of the Yelick and Kohn laboratories for their expert assistance and guidance. The authors would also like to acknowledge the considerable expertise of Stacey Piotrowski in conducting the rabbit mandible surgeries.”

The authors apologize for these errors and state that this does not change the scientific conclusions of the article in any way. The original article has been updated.
