# Supplementary material for: Use of Human Dental Pulp and Endothelial Cell Seeded Tyrosine-Derived Polycarbonate Scaffolds for Robust in vivo Alveolar Jaw Bone Regeneration
Source: Front Bioeng Biotechnol. 2020 Jul 17;8:796. doi: 10.3389/fbioe.2020.00796 (PMC7380083; doi:10.3389/fbioe.2020.00796)
Supplement: TABLE S1 — Micro-CT analyses of Bone Density and Bone Volume showed no difference between accellular and cell seeded samples. [file Table_1.pdf]

		Bone Density (mean±SD)	Bone volume (BV) mm^3
1M	Accellular (Jaw 3)	0.37±0.24	149.17
	Accellular (Jaw 5)	0.54±0.07	51.73
	Cells (Jaw 4)	0.48±0.09	53.16
	Cells (Jaw 7)	0.60±0.08	20.38
	Cells (Jaw 9)	0.55±0.08	55.88
3M	Accellular (Jaw 8)	0.40±0.10	115.47
	Cells (Jaw 2)	0.55±0.25	61.09
	Cells (Jaw 6)	0.55±0.23	162.51
	Cells (Jaw 10)	0.45±0.06	247.06
	Control (Jaw 10)	0.42±0.23	250.44
